# OPPORTUNITIES, FEASIBILITY, AND CHALLENGES OF USING IMMERSIVE VIRTUAL REALITY IN AGING RESEARCH

**DOI:** 10.1093/geroni/igac059.2414

**Published:** 2022-12-20

**Authors:** Cornelia Wrzus

**Affiliations:** Ruprecht Karls University Heidelberg, Heidelberg, Baden-Wurttemberg, Germany

## Abstract

Immersive virtual reality (iVR), that is, 3D-scenarios presented in head-mounted displays, is rarely used in aging research, although it gained much popularity recently in medical, educational, consumer, and gaming contexts and offers advantages such as real-life scenarios under experimental control. Still, iVR might be less suited for older adults, if they do not experience realism (i.e., presence) or feel strong cyber-sickness (e.g., nausea, headaches). The current preregistered project (osf.io/ryz2c) examined the opportunities, feasibility, and challenges of immersive Virtual Reality for studying age differences in socio-emotional processes. Up to now, 50 younger (age M = 23.5) and 50 older adults (age M = 67.9) saw different socio-emotional situations in iVR using a HTCvive headset, which included eye tracking, and then rated experiences of presence and cyber-sickness. Results showed that feelings of being present in the virtual reality were moderate to high, while cyber-sickness was generally low, and did not differ significantly between younger and older adults. Further analyses explore associations with technology acceptance, health, and personality. The findings suggest that using iVR with older adults is feasible, and creates similar levels of realism and low cyber-sickness as among young adults. The discussion highlights the opportunities and challenges of using iVR for studying age differences in cognitive, emotional, or social processes in experimentally controlled, real-life scenarios.

